# A Machine Learning-Based Analytic Pipeline Applied to Clinical and Serum IgG Immunoproteome Data To Predict Chlamydia trachomatis Genital Tract Ascension and Incident Infection in Women

**DOI:** 10.1128/spectrum.04689-22

**Published:** 2023-06-15

**Authors:** Chuwen Liu, Neha Vivek Mokashi, Toni Darville, Xuejun Sun, Catherine M. O’Connell, Katrin Hufnagel, Tim Waterboer, Xiaojing Zheng

**Affiliations:** a Department of Biostatistics, University of North Carolina at Chapel Hill, Chapel Hill, North Carolina, USA; b Department of Pediatrics, University of North Carolina at Chapel Hill, Chapel Hill, North Carolina, USA; c Infections and Cancer Epidemiology, German Cancer Research Center (Deutsches Krebsforschungszentrum), Heidelberg, Germany; Nanchang University

**Keywords:** ascension, biomarker, *Chlamydia* genital tract infection, incident infection, machine learning, pipeline

## Abstract

We developed a reusable and open-source machine learning (ML) pipeline that can provide an analytical framework for rigorous biomarker discovery. We implemented the ML pipeline to determine the predictive potential of clinical and immunoproteome antibody data for outcomes associated with Chlamydia trachomatis (*Ct*) infection collected from 222 cis-gender females with high *Ct* exposure. We compared the predictive performance of 4 ML algorithms (naive Bayes, random forest, extreme gradient boosting with linear booster [xgbLinear], and *k*-nearest neighbors [KNN]), screened from 215 ML methods, in combination with two different feature selection strategies, Boruta and recursive feature elimination. Recursive feature elimination performed better than Boruta in this study. In prediction of *Ct* ascending infection, naive Bayes yielded a slightly higher median value of are under the receiver operating characteristic curve (AUROC) 0.57 (95% confidence interval [CI], 0.54 to 0.59) than other methods and provided biological interpretability. For prediction of incident infection among women uninfected at enrollment, KNN performed slightly better than other algorithms, with a median AUROC of 0.61 (95% CI, 0.49 to 0.70). In contrast, xgbLinear and random forest had higher predictive performances, with median AUROC of 0.63 (95% CI, 0.58 to 0.67) and 0.62 (95% CI, 0.58 to 0.64), respectively, for women infected at enrollment. Our findings suggest that clinical factors and serum anti-*Ct* protein IgGs are inadequate biomarkers for ascension or incident *Ct* infection. Nevertheless, our analysis highlights the utility of a pipeline that searches for biomarkers and evaluates prediction performance and interpretability.

**IMPORTANCE** Biomarker discovery to aid early diagnosis and treatment using machine learning (ML) approaches is a rapidly developing area in host-microbe studies. However, lack of reproducibility and interpretability of ML-driven biomarker analysis hinders selection of robust biomarkers that can be applied in clinical practice. We thus developed a rigorous ML analytical framework and provide recommendations for enhancing reproducibility of biomarkers. We emphasize the importance of robustness in selection of ML methods, evaluation of performance, and interpretability of biomarkers. Our ML pipeline is reusable and open-source and can be used not only to identify host-pathogen interaction biomarkers but also in microbiome studies and ecological and environmental microbiology research.

## INTRODUCTION

An important question when investigating host microbe interactions is how to best identify biomarkers predictive of infection risk or outcome(s). Machine learning (ML) models are becoming popular algorithms for biomarker discovery (1–4). Four major challenges exist for identifying predictors by ML in high-dimensionality data. First is the selection of an optimal ML algorithm, given the wide and ever-increasing variety of ML approaches being developed and refined. Frequently, studies have arbitrarily selected a single ML approach, which may not be appropriate for the data set, since the performance of specific algorithms on different data sets can vary greatly. Thus, no universal best approach exists. Second, it can be difficult to identify the correct feature selection strategies to reduce dimensionality. Feature selection, used to identify the most relevant features in building a useful and constructive prediction model, is a key step in ML that can greatly influence prediction performance and prevent overfitting (5). ML methods risk achieving good model fit with training data using details that happen by chance and thus are irrelevant features. This “overfitting” will negatively impact the subsequent performance of the model when it is applied to new, unseen data. Two state-of-the-art feature selection procedures, recursive feature elimination (RFE) (6) and Boruta (7), have displayed variable performance (8–11), depending on the data sets to which they have been applied. Third, opportunities for validation of candidate biomarkers are frequently limited. The gold standard is to replicate study findings in independent large cohorts. However, it can be difficult to recruit a validation cohort within a feasible timescale. A robust internal validation approach is needed. Finally, ML algorithms are criticized as black boxes due to the complexity of models, which may compromise interpretability. Biological interpretation of selected features can generate insight into the underlying mechanism and guide experimental design, ultimately yielding confirmatory data that validate or exclude potential biomarkers.

To address these challenges, we developed a reusable open-source ML pipeline, which enables systematic screening of data set-appropriate ML algorithms from over 200 ML algorithms. The pipeline identifies the optimal feature selection strategy by comparing the most commonly used feature selection procedures, evaluates reliability of the predictive capacity of biomarkers using a resampling approach, and interprets the roles of the biomarkers on prediction by quantifying the importance of features and measuring their effects on altering the risk of the outcome. The goal of this study was to provide an analytical framework and guideline that can be applied to host-microbe studies, especially those with high-throughput data, for rigorous biomarker discovery and functional interpretation.

We generated a case study by applying this ML pipeline to prediction of outcomes of Chlamydia trachomatis (*Ct*) infection, using clinical and immunoproteome antibody data collected from a unique and well-defined longitudinal cis-gender female cohort with high risk of *Ct* exposure and endometrial *Ct* infection status determined by analysis of biopsy specimens (12, 13). *Ct* infection is the leading bacterial sexually transmitted infection in the United States. Infection is often asymptomatic, resulting in absence or delay of treatment. In up to 50% of infected women, untreated *Ct* may ascend from the cervix to the uterus and Fallopian tubes and cause severe reproductive morbidities, including pelvic inflammatory disease (PID), chronic pelvic pain, infertility, and ectopic pregnancy. Repeated infections are common and may worsen disease. Noninvasive biomarkers for ascension of *Ct* to the upper female genital tract could serve as correlative endpoints for *Ct* vaccine efficacy in clinical trials, and biomarkers of enhanced risk of reinfection could identify individuals likely to benefit from increased screening to prevent sequelae (14). Previously, we found that the indirect effects of serum and cervical IgG against whole *Ct* reduced cervical *Ct* burden, a mediator for ascending infection, but their direct effects negated any protective role for IgG in prevention of *Ct* ascension (15). In addition, increased levels of IgG against whole *Ct* were associated with significantly increased risk for incident infection (15). *Ct* expresses over 875 proteins (16). Applying logistic regression to serum IgG-binding data from a whole-proteome *Ct* array that screened serum pools from *Ct*-exposed women, we identified antibodies to some *Ct* proteins that weakly associated with decreased risk of ascension and others associated with increased risk for incident infection (13). In addition, we previously determined select sociodemographic, coinfection, and behavioral risk factors contributing to ascending and incident infection (12). However, we had not systematically studied the predictive capability of these risk factors and anti-*Ct* protein-specific antibodies as biomarkers. Analysis of these data using our ML pipeline revealed that chlamydial-antigen-specific antibodies and risk factors are insufficient to serve as biomarkers for ascension or incident infection.

## RESULTS

### Study population and design.

Data from 222 women, 144 infected and 78 uninfected, were used in this study. The sociodemographic characteristics of participants have been described previously (13). Women in this study were young (median age, 21 years; range, 18 to 35 years), and the majority of them were single, were African American, and were high school graduates or had some college education. For ascension analysis, women testing positive for cervical and endometrial infection were defined as Endo+ (*n* = 77), while those testing positive for cervical infection only were defined as Endo− (*n* = 67). For incident infection analysis, women having *Ct* at any follow-up visit or reporting *Ct* infection between visits were defined as F/U+. Women who completed at least 3 follow-up visits over the course of a year without detected/reported CT infection were defined as F/U−. Among the women with *Ct* infection at enrollment, 47 (41%) were F/U+ and 69 (59%) were F/U−. Among women without *Ct* infection at enrollment, 9 (18%) were F/U+ and 42 (82%) were F/U−. Seropositivity profiles of 121 *Ct* protein-specific serum IgGs (13), previously identified clinical risk factors for ascension (oral contraceptives and gonorrhea), and incident infection (gonorrhea, age, cervical infection at enrollment, and sex with new, uncircumcised, or infected partners) (12) were subjected to biomarker analysis.

**FIG 1:**
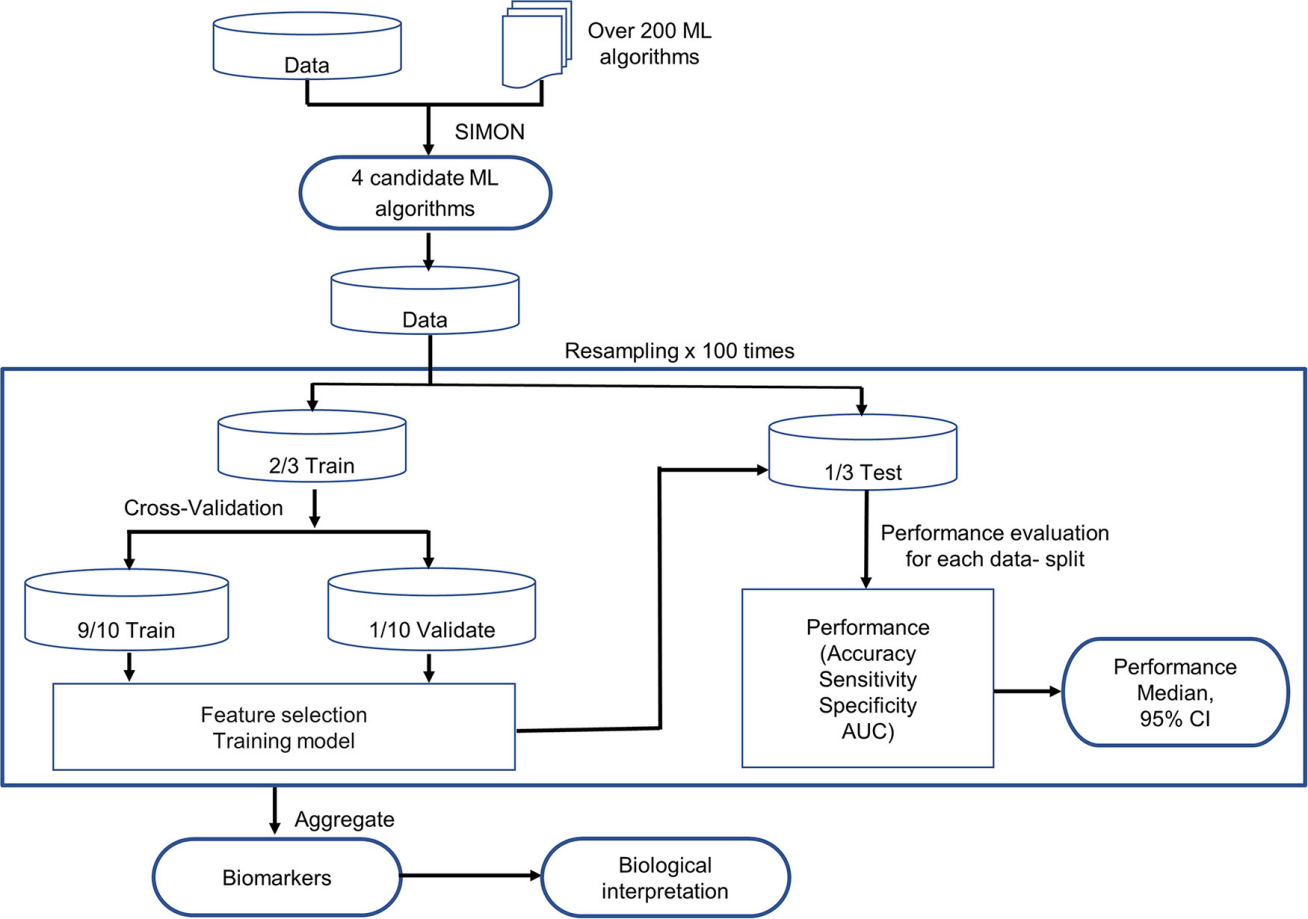
Workflow of the resampling-based ML pipeline. We first selected 4 candidate ML algorithms using SIMON. We then split the data to create a training set (2/3) and held-out test set (1/3). We performed 10-fold cross-validation on the training data to train the models by identifying the key features through feature selection and generating prediction models by ML algorithms. Performance of the training model was evaluated on the held-out test data. We repeated the whole process 100 times to generate the median and CI of performance and determine the final biomarkers. We eventually provided biological interpretation of the biomarkers.

We established a resampling-based ML pipeline for selection of the optimal ML algorithm, determination of feature selection method, identification of biomarkers, evaluation of prediction performance, and interpretation of biomarkers, which is illustrated in Fig. 1.

### Identification of 4 candidate ML algorithms from over 200 ML algorithms.

We used Sequential Iterative Modeling “OverNight” (SIMON) (17) to systematically screen for candidate ML algorithms from 215 different algorithms. Table S1 in the supplemental material lists results of the top 10 algorithms with best prediction accuracy for ascension and incident infection. Four algorithms, including naive Bayes, random forest, extreme gradient boosting with linear booster (xgbLinear), and *k*-nearest neighbors (KNN), were consistently among the top 10 performers for ascension and incident infection. These 4 methods were selected for further analysis and are briefly described in Table 1.

**TABLE 1:** Description of the 4 ML methods

Method	Description
*k*-nearest neighbors	A nonparametric machine learning algorithm without assumptions about data. It classifies new data points based on how its nearest *k* neighbors (data points) are classified in the feature space
Extreme gradient boosting with linear booster	Combines multiple single prediction models with different weights; higher weights are assigned to the single models with higher cross-validated prediction accuracy in the training data set
Random forest	An ensemble of multiple-decision trees to minimize the impact of individual errors of trees on final prediction
Naive Bayes	A conditional probabilistic classification model with independence assumptions between the features

### RFE performed better than Boruta for feature selection.

Recursive feature elimination (RFE) and Boruta are two feature selection methods that were used in our prediction procedure.

**FIG 2:**
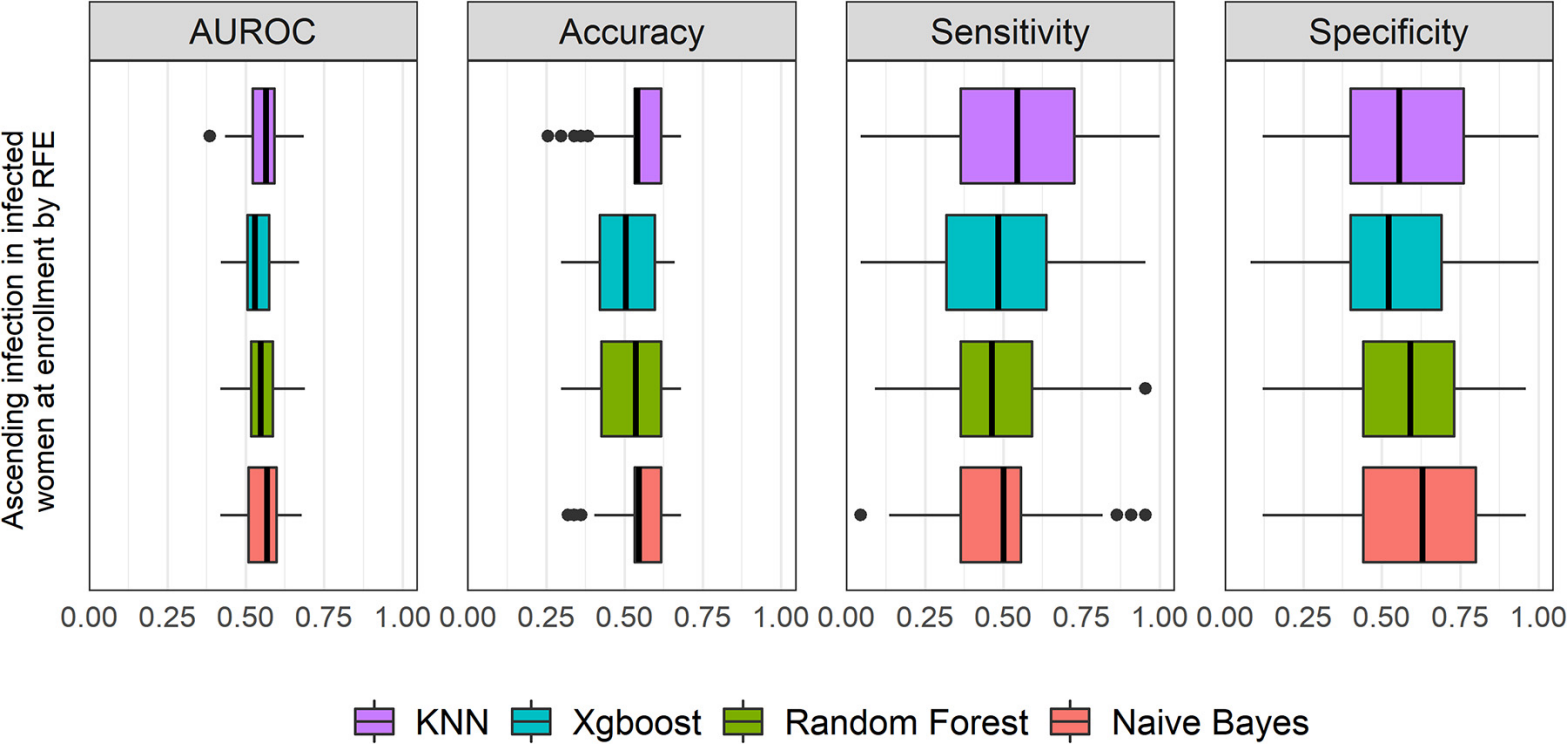
Graphic summary of the prediction performance of 4 ML algorithms for ascension by RFE feature selection method. The performances of the AUROC, sensitivity, and specificity among all algorithms were not significantly different from each other, with all *P* values being >0.05 by Mood’s median test. The performances across all methods were low, with the best median AUROC, accuracy, and specificity (0.57, 0.55, and 0.63, respectively) exhibited by naive Bayes. The box plot shows quartiles at the box ends and the median as the vertical line in the box. The whiskers show the farthest points that were not outliers. Outliers were defined as data points that are not within 1.5 times the interquartile ranges.

**FIG 3:**
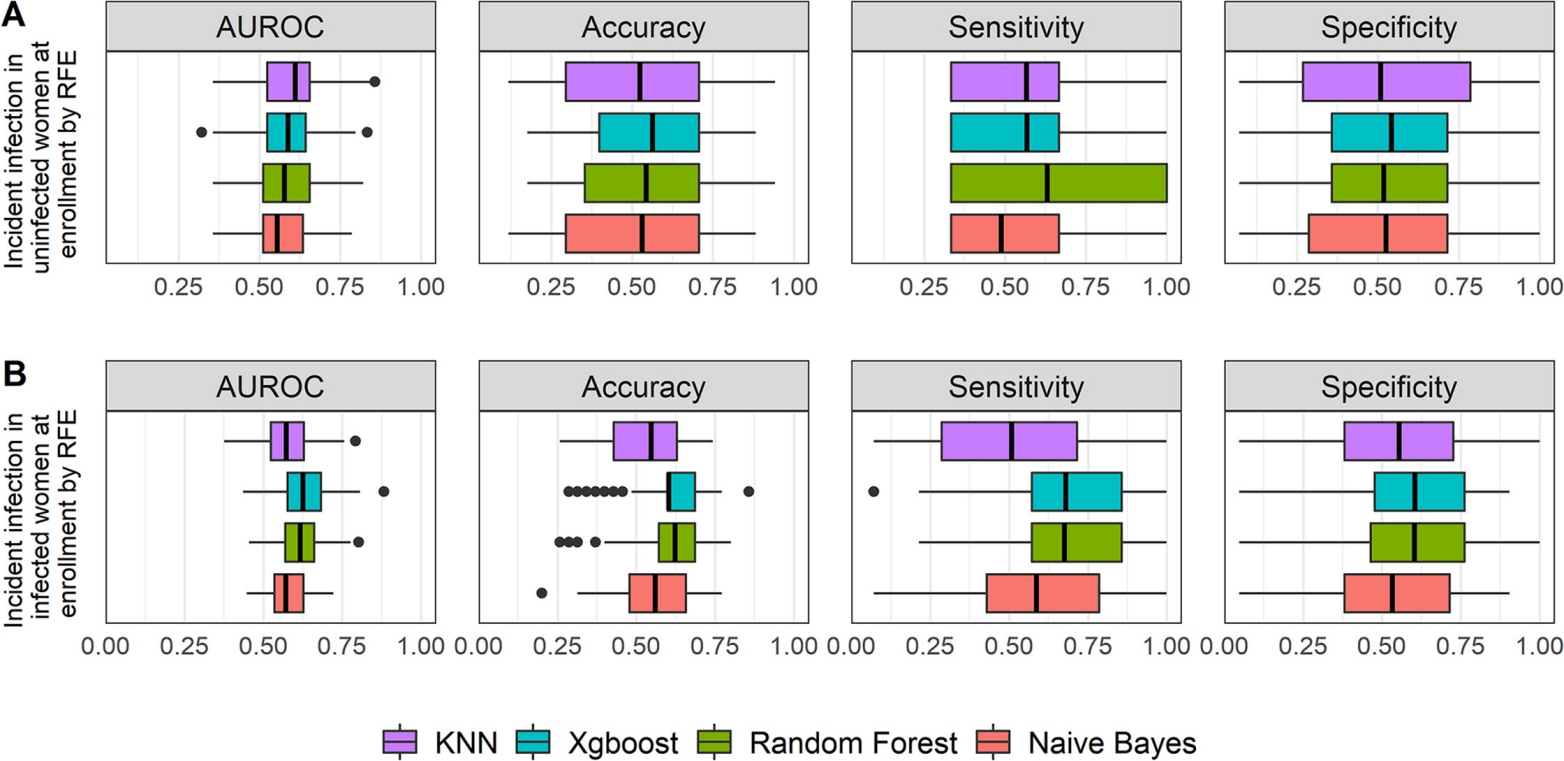
Graphic summary of the prediction performance of 4 ML algorithms for incident infection by the RFE feature selection method. For uninfected women at enrollment (A), the performances of AUROC, accuracy, sensitivity, and specificity among all algorithms were not significantly different from each other, with all *P* values being >0.05 by Mood’s median test. The performances across all methods were low, with the best median AUROC of 0.61 obtained with KNN, the best median value of accuracy of 0.56 obtained with xgbLinear, and the best median value of sensitivity of 0.63 obtained with random forest. For infected women at enrollment (B), the predictive performances of random forest and xgbLinear were significantly higher than those of other ML models (all *P* values < 0.05 by Mood’s median test), with median AUROC of 0.62 and 0.63, respectively, accuracy of 0.62 and 0.60, respectively, sensitivity of 0.68 (both), and specificity of 0.60 (both). The performances of naive Bayes and *k*-nearest neighbors were not significantly different from one another. The box plot shows quartiles at the box ends and the median as the vertical line in the box. The whiskers show the farthest points that were not outliers. Outliers were defined as data points that are not within 1.5 times the interquartile ranges.

**(i) Performance of 4 candidate ML algorithms with RFE feature selection in prediction of *Ct* ascension and incident infection.** We first applied the resampling-based RFE procedure to compare the prediction performance of the selected 4 algorithms. RFE is a backward feature selection method which searches for a subset of features by starting with all features in the training data set and then removing features until a specified number remains. We randomly partitioned the data into 2/3 training and 1/3 test sets and repeated this process 100 times to generate 100 training/test data sets. In each training data set, leveraging statistical cross-validation, we conducted RFE to tune the parameters, rank the importance of features, and determine the best subset of features with the smallest prediction error to generate a prediction model, which was evaluated in the resampled independent test data to determine performance of prediction. The performance was assessed by area under the receiver operating characteristic curve (AUROC), accuracy (correct classification rate), sensitivity (true positive rates of ascension or reinfection), and specificity (true negative rates). This procedure was repeated 100 times. The prediction performances of 4 candidate methods for ascension and incident infection are summarized in Fig. 2 and 3 and Tables 2 to 4. Since the performance had tied values, we used the true-median method, which is a well-established method for calculation of medians for data with tied values (18).

**TABLE 2:** Performance of 4 methods in ascension with RFE feature selection

Method	AUC	Accuracy	Sensitivity	Specificity
*k*-nearest neighbors	0.56 (0.54, 0.59)	0.54 (0.44, 0.64)	0.54 (0.46, 0.64)	0.56 (0.47, 0.68)
Extreme gradient boosting with linear booster	0.53 (0.51, 0.55)	0.50 (0.41, 0.60)	0.48 (0.39, 0.58)	0.52 (0.43, 0.66)
Random forest	0.55 (0.52, 0.59)	0.54 (0.43, 0.62)	0.46 (0.34, 0.57)	0.59 (0.49, 0.69)
Naive Bayes	0.57 (0.54, 0.59)	0.55 (0.45, 0.66)	0.50 (0.36, 0.60)	0.63 (0.53, 0.74)

**TABLE 3:** Performance of 4 methods in reinfection in uninfected women at enrollment with RFE feature selection

Method	AUC	Accuracy	Sensitivity	Specificity
*k*-nearest neighbors	0.61 (0.49, 0.70)	0.52 (0.41, 0.61)	0.57 (0.45, 0.67)	0.51 (0.40, 0.60)
Extreme gradient boosting with linear booster	0.59 (0.46, 0.71)	0.56 (0.44, 0.64)	0.57 (0.46, 0.67)	0.54 (0.43, 0.65)
Random forest	0.58 (0.48, 0.69)	0.54 (0.43, 0.66)	0.63 (0.53, 0.74)	0.52 (0.40, 0.64)
Naive Bayes	0.56 (0.48, 0.69)	0.53 (0.41, 0.63)	0.49 (0.37, 0.59)	0.53 (0.39, 0.62)

**TABLE 4:** Performance of 4 methods for reinfection in infected women at enrollment with RFE feature selection

Method	AUC	Accuracy	Sensitivity	Specificity
*k*-nearest neighbors	0.57 (0.53, 0.63)	0.55 (0.43, 0.62)	0.51 (0.42, 0.64)	0.55 (0.44, 0.65)
Extreme gradient boosting with linear booster	0.63 (0.58, 0.67)	0.60 (0.51, 0.71)	0.68 (0.55, 0.77)	0.60 (0.48, 0.70)
Random forest	0.62 (0.58, 0.64)	0.62 (0.51, 0.74)	0.68 (0.56, 0.77)	0.60 (0.47, 0.68)
Naive Bayes	0.57 (0.52, 0.62)	0.56 (0.47, 0.68)	0.59 (0.50, 0.73)	0.53 (0.46, 0.65)

For ascension (Fig. 2 and Table 2), the predictive performance of naive Bayes was slightly higher though not significantly different than other algorithms with a median AUROC of 0.57 (95% confidence interval [CI], 0.54 to 0.59) (*P* = 0.52 by Mood’s median test). Similarly, random forest, xgbLinear, and *k*-nearest neighbors AUROC values were not significantly different from one another and had medians of 0.55 (95% CI, 0.52 to 0.59), 0.53 (95% CI, 0.51 to 0.55), and 0.56 (95% CI, 0.54 to 0.59), respectively. The median accuracy and specificity of naive Bayes were also slightly higher, with values of 0.55 and 0.63, respectively (*P* = 0.89 and 0.07 by Mood’s median test).

For incident infection during follow-up, we stratified our analysis into separate evaluations among uninfected and infected women at enrollment. In uninfected women at enrollment (Fig. 3A and Table 3), performances of AUROC, accuracy, sensitivity, and specificity among all algorithms were not significantly different, with all *P* values being >0.05 by Mood’s median test. The KNN had slightly better performance than other algorithms with a median AUROC of 0.61 (95% CI, 0.49 to 0.70). The accuracies across all methods were low, with the best median value of accuracy of 0.56 predicted by xgbLinear and the best median value of sensitivity being 0.63 by random forest.

For incident infection among infected women at enrollment (Fig. 3B and Table 4), xgbLinear and random forest had higher performance, with median AUROC of 0.63 (95% CI, 0.58 to 0.67) and 0.62 (95% CI, 0.58 to 0.64). They were not significantly different from one another but were significantly higher than values from other algorithms (*P* = 4.6E−02 by Mood’s median test). They also had higher accuracies, with median values of 0.62 and 0.60, respectively, which was due to the higher median values of sensitivity 0.68 for both.

### (ii) Performance of 4 candidate ML algorithms with Boruta feature selection method for ascension and incident infection.

We next applied the resampling-based Boruta feature selection method to the selected 4 ML algorithms and compared their performance to that obtained with RFE feature selection. The resampling procedure was as described above.

**FIG 4:**
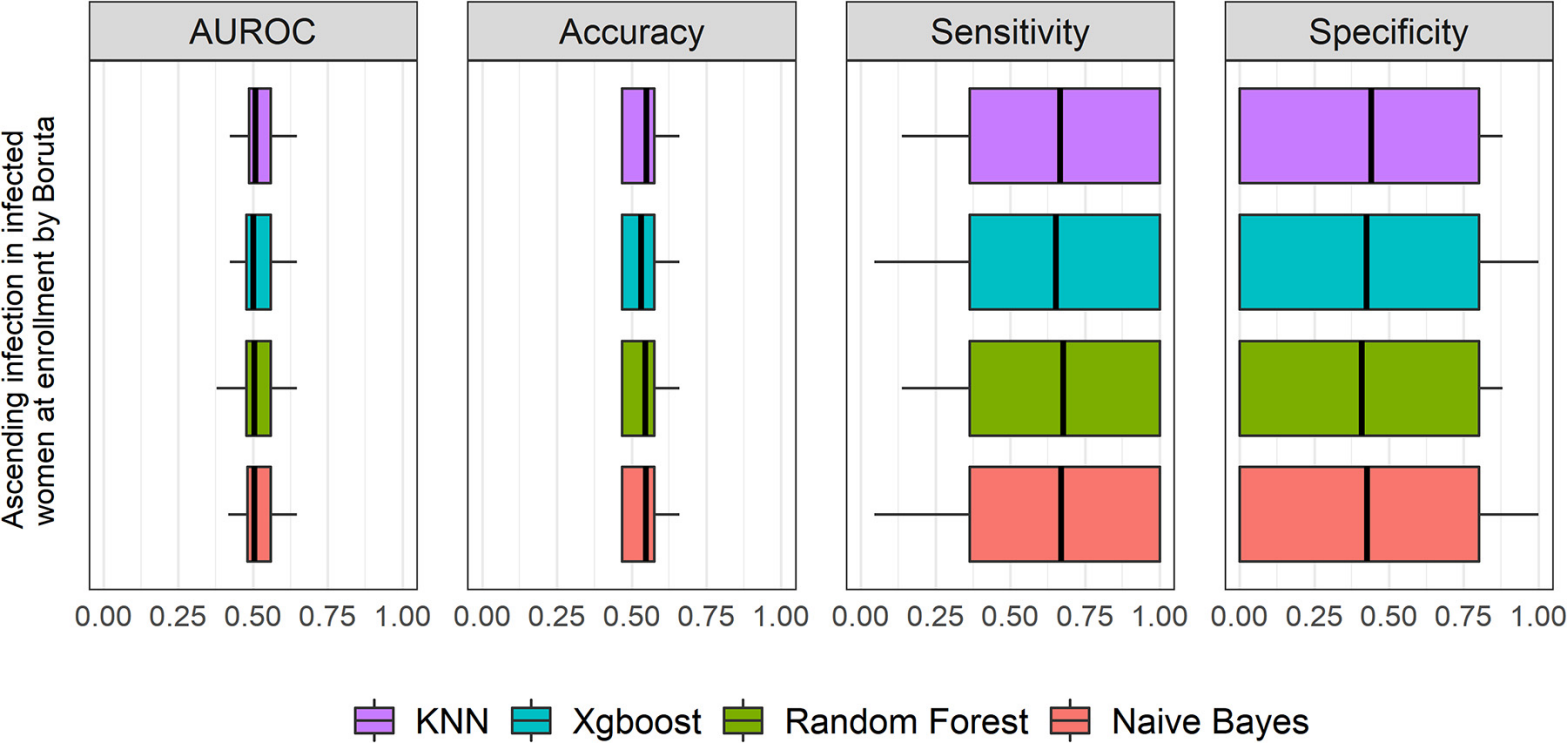
Graphic summary of the prediction performance of 4 ML algorithms for ascension by the Boruta feature selection method. The performances of AUROC, accuracy, sensitivity, and specificity among all algorithms were not significantly different from each other, with all *P* values being >0.05 by Mood’s median test. The performances across all methods were very low, with median AUROC of 0.5 to 0.51, accuracy of 0.53 to 0.55, sensitivity of 0.65 to 0.68, and specificity of 0.41 to 0.44, respectively. The box plot shows quartiles at the box ends and the median as the vertical line in the box. The whiskers show the farthest points that were not outliers. Outliers were defined as data points that are not within 1.5 times the interquartile ranges.

For ascension (Fig. 4 and Table 5), the predictive performances of the 4 algorithms were very low, with a median AUROC of 0.5 to 0.51 (95% CI, 0.48 to 0.53), and not significantly different from each other (*P* = 0.99 by Mood’s median test). The median accuracy values among the 4 methods were around 0.53 to 0.55, with median sensitivities of 0.65 to 0.68 and median specificities of 0.41 to 0.44.

**TABLE 5:** Performance of 4 methods in ascension with Boruta feature selection

Method	AUC	Accuracy	Sensitivity	Specificity
*k*-nearest neighbors	0.51 (0.49, 0.53)	0.55 (0.43, 0.66)	0.67 (0.50, 0.80)	0.44 (0.31, 0.54)
Extreme gradient boosting with linear booster	0.50 (0.48, 0.53)	0.53 (0.39, 0.66)	0.65 (0.51, 0.79)	0.42 (0.29, 0.54)
Random forest	0.50 (0.49, 0.53)	0.54 (0.39, 0.65)	0.68 (0.52, 0.82)	0.41 (0.28, 0.54)
Naive Bayes	0.50 (0.48, 0.53)	0.55 (0.42, 0.66)	0.67 (0.52, 0.79)	0.42 (0.28, 0.54)

**FIG 5:**
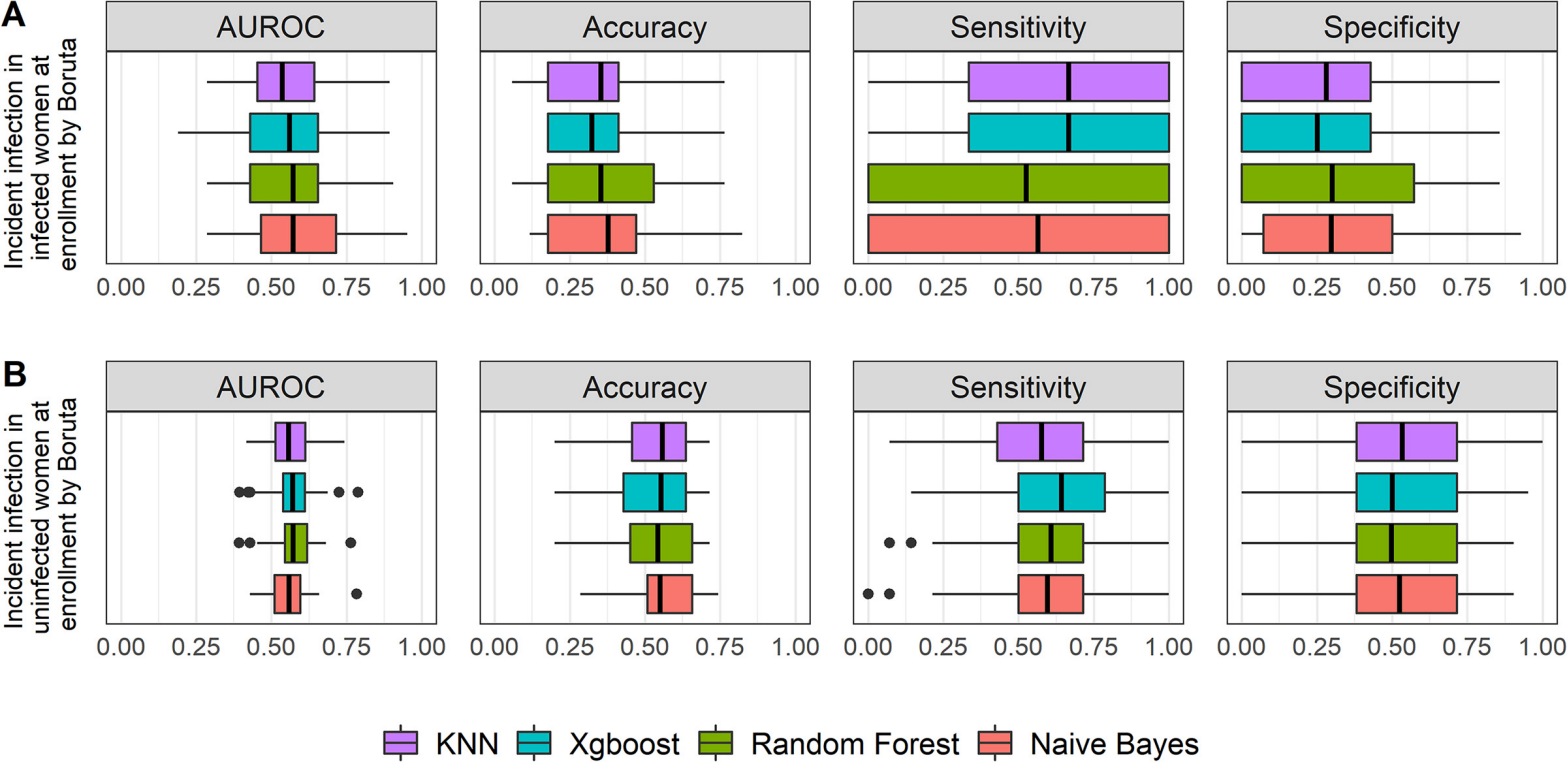
Graphic summary of the prediction performance of 4 ML algorithms for incident infection by the Boruta feature selection method. For uninfected women at enrollment (A), the performances across all methods were very low, with median AUROC of 0.54 to 0.56, accuracy of 0.32 to 0.38, sensitivity of 0.53 to 0.67, and specificity of 0.25 to 0.30. For infected women at enrollment (B), the performances of AUROC, accuracy, sensitivity, and specificity among all algorithms were not significantly different from each other, with all *P* values being >0.05 by Mood’s median test. The performances across all methods were low, with median AUROC of 0.56 to 0.57, accuracy of 0.54 to 0.56, sensitivity of 0.58 to 0.64, and specificity of 0.50 to 0.53. The box plot shows quartiles at the box ends and the median as the vertical line in the box. The whiskers show the farthest points that were not outliers. Outliers were defined as data points that are not within 1.5 times the interquartile ranges.

For incident infection during follow-up, in women who were uninfected at enrollment (Fig. 5A and Table 6), the predictive performances of 4 algorithms were similar, with a median AUROC of 0.54 to 0.56, and were not significantly different from each other (*P* = 0.89 by Mood’s median test). The 4 methods had very low median values of accuracy, 0.32 to 0.38, which were driven by the low median values of specificity, 0.25 to 0.30.

**TABLE 6:** Performance of 4 methods in reinfection in uninfected women at enrollment with Boruta feature selection

Method	AUC	Accuracy	Sensitivity	Specificity
*k*-nearest neighbors	0.54 (0.45, 0.67)	0.35 (0.21, 0.50)	0.67 (0.47, 0.87)	0.28 (0.10, 0.45)
Extreme gradient boosting with linear booster	0.56 (0.43, 0.66)	0.32 (0.20, 0.49)	0.67 (0.46, 0.85)	0.25 (0.10, 0.43)
Random forest	0.56 (0.49, 0.68)	0.35 (0.21, 0.49)	0.53 (0.28, 0.73)	0.30 (0.13, 0.48)
Naive Bayes	0.56 (0.48, 0.73)	0.38 (0.20, 0.50)	0.56 (0.32, 0.79)	0.30 (0.13, 0.46)

For incident infection among women who were infected at enrollment (Fig. 5B and Table 7), xgbLinear and random forest had higher performance, with median AUROC of 0.57 (95% CI, 0.54 to 0.61) and 0.57 (95% CI, 0.53 to 0.61), respectively. They were not significantly different from other algorithms (*P* = 0.93 by Mood’s median test). They also had higher sensitivity, with median values of 0.64 and 0.61, respectively, which were nevertheless not significantly different from those of the other algorithms (*P* = 0.27 by Mood’s median test).

**TABLE 7:** Performance of 4 methods in reinfection in infected women at enrollment with Boruta feature selection

Method	AUC	Accuracy	Sensitivity	Specificity
*k*-nearest neighbors	0.56 (0.52, 0.60)	0.56 (0.41, 0.67)	0.58 (0.46, 0.71)	0.53 (0.40, 0.66)
Extreme gradient boosting with linear booster	0.57 (0.54, 0.61)	0.55 (0.40, 0.69)	0.64 (0.50, 0.73)	0.50 (0.35, 0.63)
Random forest	0.57 (0.53, 0.61)	0.54 (0.42, 0.68)	0.61 (0.48, 0.73)	0.50 (0.40, 0.67)
Naive Bayes	0.56 (0.48, 0.61)	0.55 (0.43, 0.69)	0.60 (0.50, 0.72)	0.52 (0.38, 0.63)

All these findings suggested that predictive performance of Boruta for ascension and incident infection was inferior to that of the RFE feature selection method. We thus used RFE feature selection strategy for further analyses in this study.

### Determination and interpretation of biomarkers by 4 candidate ML algorithms with RFE.

To identify optimal and stable biomarkers with each algorithm, we aggregated data from 100 training sets, averaged the variable importance scores of each feature from 100 training sets and reranked them. Using RFE and statistical cross validation, the best subset of features with the smallest error was selected as the final panel of biomarkers, which are listed in descending order of importance in Table 8. Biomarkers identified by at least three of four ML algorithms were considered consensus biomarkers.

**TABLE 8:** Biomarkers predictive of ascension^*a*^

*k*-nearest neighbors	Extreme gradient boosting with linear booster	Random forest	Naive Bayes
**Pgp3**	**Pgp3**	**Pgp3**	**Pgp3**
**CT123**	CT798	**CT123**	**CT123**
CT732	**CT123**	CT017	
CT017	CT681	CT601	
	CT242	Pills^*b*^	
	CT529	CT798	
	CT858	CT759	
	CT443	CT442	
		CT242	

a Feature importance is given in descending order. Candidate biomarkers in bold were selected by at least 3 ML algorithms.

b Oral birth control pills.

**FIG 6:**
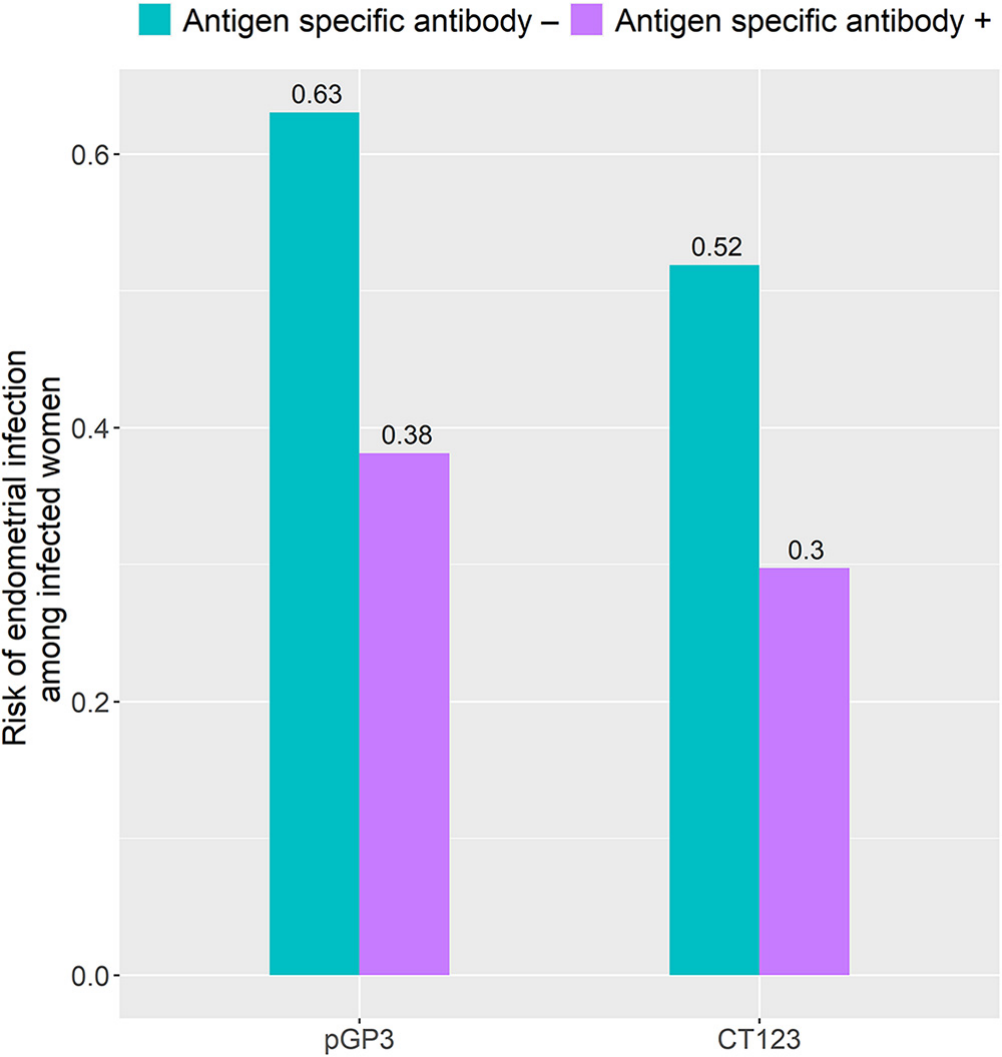
Seropositivity to Pgp3 and CT123 were associated with decreased risk of endometrial infection. The risks of ascension were significantly higher in women with negative antibody responses to Pgp3 and CT123/AccB than in those with positive responses (*P* = 0.009 and 0.016, respectively) by chi-square test. The numbers above each bar indicate the incident risk of endometrial infection among each group.

The consensus biomarkers for ascension were anti-Pgp3 (a plasmid-encoded virulence factor) and anti-CT123 (AccB; acetyl coenzyme A [acetyl-CoA] carboxylase biotin carboxyl carrier protein). The risks of ascension were significantly higher in women with negative than positive antibody responses to Pgp3 and CT123/AccB (*P* = 0.009 and 0.016, respectively [Fig. 6]), suggesting that these two antibodies could play a role in protection from ascending infection.

**FIG 7:**
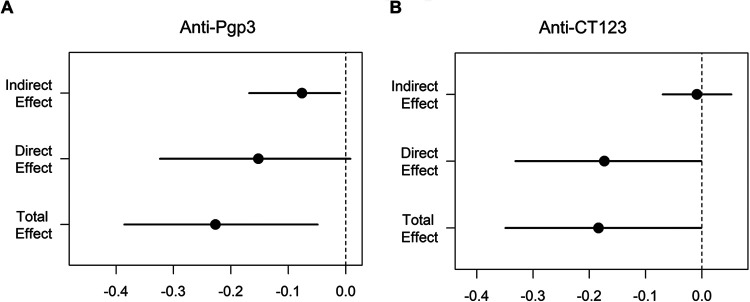
Causal mediation analysis revealed that serum anti-Pgp3 (A) and anti-CT123 (B) decreased the risk of ascension through indirect and direct effects, respectively. The total effect of anti-Pgp3 on reducing the risk of ascension was significant (*P* = 0.008), driven by a significant indirect effect mediated through reducing burden (*P* = 0.024) and marginally significant direct effect (*P* = 0.068). The direct effect of anti-CT123/AccB on reducing the risk of ascension was significant (*P* = 0.049) but was negated slightly by an insignificant indirect effect, leading to a borderline significant total effect (*P* = 0.06). The *x* axis represents the effect value; each dot represents the point estimate, and each line represents the confidence interval. The dotted vertical line indicates absence of an effect of antibody on ascension.

To further explore the functional interpretation of biomarkers predicting ascension, we conducted a causal mediation analysis (19) which decomposed the total effect of antibody on endometrial infection into two parts, direct and indirect. The direct effect is the effect of antibody on ascension absent the mediator of cervical *Ct* burden, while the indirect effect is the effect of antibody on ascension through the mediator. The total effect of anti-Pgp3 on reducing the risk of ascension was significant (*P* = 0.008) (Table 9 and Fig. 7A), driven by significant indirect effect mediated through reducing burden (*P* = 0.024) and marginally significant direct effect (*P* = 0.068). The direct effect of anti-CT123/AccB on reducing the risk of ascension was significant (*P* = 0.049) but was negated slightly by an insignificant indirect effect, leading to a borderline significant total effect (*P* = 0.06) (Table 9 and Fig. 7B).

**TABLE 9:** Effects of anti-Pgp3 and -CT123 on probability of Chlamydia ascension to the endometrium determined by causal mediator analysis

Antibody	Indirect effect or causal mediator effect through burden		Direct effect independent of burden		Total effect	
	Avg effect (95% CI)^*a*^	*P*	Avg effect (95% CI)^*a*^	*P*	Avg effect (95% CI)^*a*^	*P*
Anti-Pgp3	−0.076 (−0.168, −0.01)	0.024	−0.152 (−0.324, 0.008)	0.068	−0.227 (−0.386, −0.049)	0.008
Anti-CT123	−0.005 (−0.06, 0.05)	0.892	−0.17 (−0.34, −0.001)	0.049	−0.176 (−0.34, 0.001)	0.06

a Each value represents the percent decreased (negative values) or increased (positive values) probability of ascension (percent). For example, there is a 7.6% decreased risk of ascension for women with a specific serum anti-Pgp3 IgG response.

**FIG 8:**
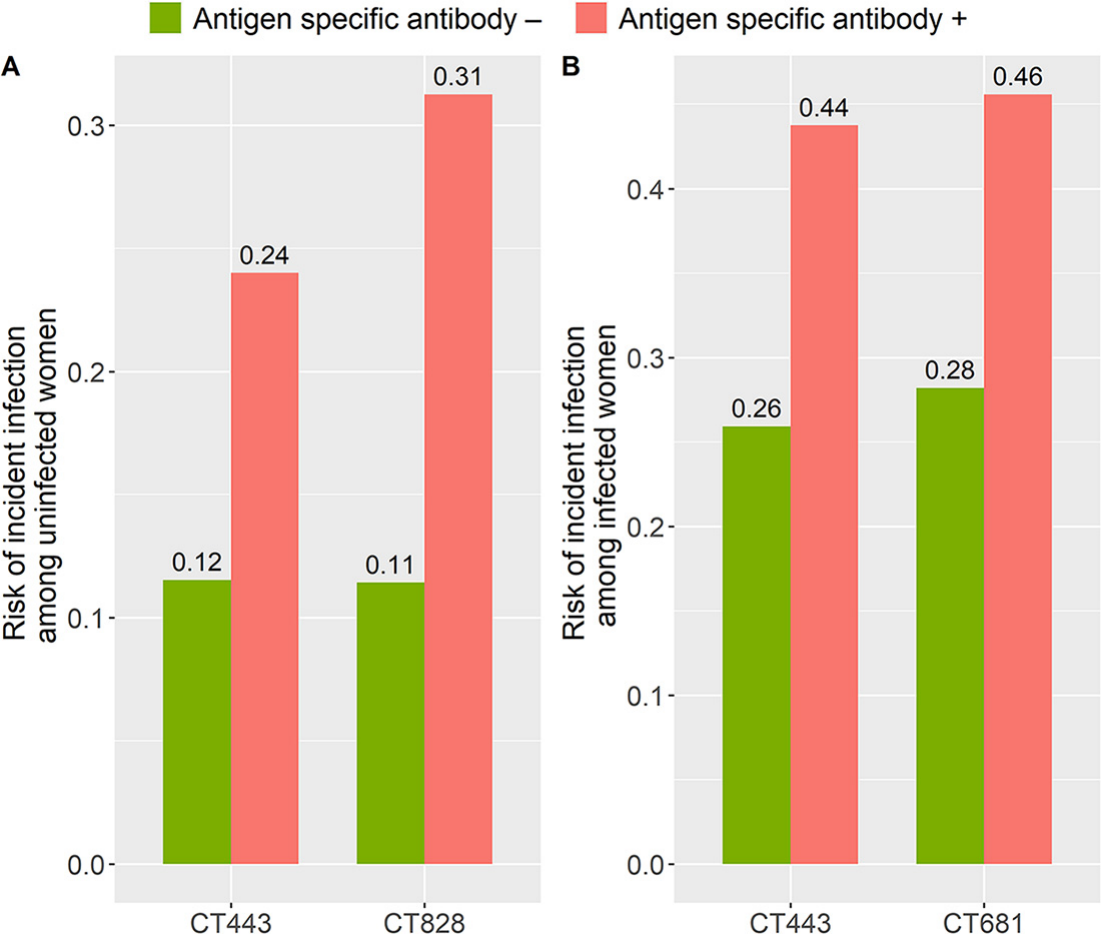
Among uninfected women at enrollment (A), seropositivity to CT443 and CT828 were associated with increased risk of incident infection. The risks of incident infection were higher in women with positive antibody responses to anti-CT443 and -CT828 than in those with negative responses, though the differences were insignificant (*P* = 0.24 and 0.08, respectively). Among infected women at enrollment (B), seropositivity to CT443 and CT681 was associated with increased risk of incident infection. The risks of incident infection were marginally significantly higher in women with positive antibody responses to anti-CT443 and -CT681 than in those with negative responses (*P* = 0.07 and 0.048, respectively) by chi-square test. The numbers above each bar indicate the incident risk of incident infection among each group.

For incident infection in uninfected women at enrollment, three features were identified as consensus biomarkers, including anti-CT443 (outer membrane complex protein B [OmcB]), anti-CT828 (ribonucleoside diphosphate reductase beta chain [NrdB]), and sex with a *Ct*-infected male during follow-up (Table 10). The risk of incident infection was higher in women with positive antibody responses to CT443/OmcB and CT828/NrdB, but the difference was not significant (*P* = 0.24 and 0.08, respectively [Fig. 8A]). Having sex with *Ct*-infected males during follow-up was previously determined to be significantly associated with increased risk of incident infection (12).

**TABLE 10:** Biomarkers predicting reinfection in uninfected women at enrollment^*a*^

*k*-nearest neighbors	Extreme gradient boosting with linear booster	Random forest	Naive Bayes
**Sex with *Ct*-infected male during F/U**	Age	Age	**Sex with *Ct*-infected male during F/U**
**CT828**	**Sex with *Ct*-infected male during F/U**	**Sex with *Ct*-infected male during F/U**	**CT828**
CT089		**CT828**	CT089
**CT443**		CT228	**CT443**
		CT017	CT228
		CT143	CT143
		CT123	CT841
		**CT443**	CT229
			Age

a Feature importance is given in descending order. Candidate biomarkers in boldface were selected by at least 3 ML algorithms. F/U, follow-up.

For incident infection in infected women at enrollment, five features were identified as consensus biomarkers. Sex with a *Ct*-infected male during follow-up, *Ct* infection limited to the cervix at enrollment, and lower age were associated with increased risk of incident infection, as previously described (12). The remaining two biomarkers identified were serum antibodies recognizing CT681 (OmpA; the major outer membrane protein [MOMP]) and CT443/OmcB (Table 11). OmcB and the MOMP (20–23) are immunoprevalent, stimulating antibody production in high percentages of exposed individuals, and immunodominant, evoking high levels of antibody in an individual (13). The risks of incident infection were marginally significantly higher in women with positive than negative antibody responses to CT443/OmcB and CT681/MOMP (*P* = 0.07 and 0.048, respectively [Fig. 8B]), suggesting that binding antibodies to these immunoprevalent and immunodominant *Ct* proteins indicate persons with increased rather than decreased risk of repeat infection.

**TABLE 11:** Biomarkers predicting reinfection in infected women at enrollment^*a*^

*k*-nearest neighbors	Extreme gradient boosting with linear booster	Random forest	Naive Bayes
**Age**	**Age**	**Sex with *Ct*-infected male during F/U**	**Age**
**Sex with *Ct*-infected male during F/U**	**Sex with *Ct*-infected male during F/U**	**Age**	**Sex with *Ct*-infected male during F/U**
**CT681**	***Ct* enroll**	**CT681**	**CT681**
***Ct* enroll**	Pgp3	**CT443**	***Ct* enroll**
	CT813	CT798	**CT443**
	**CT443**	CT486	CT798
	CT841	***Ct* enroll**	
	CT110	CT541	
		CT123	
		CT110	
		CT858	

a Feature importance is given in descending order. Candidate biomarkers in boldface were selected by at least 3 ML algorithms. F/U, follow-up; *Ct* enroll, site of C. trachomatis infection at enrollment, including subgroups with both cervical and endometrial infection, cervical infection only, and no infection.

## DISCUSSION

Induction of pathogen-specific adaptive responses is complex, with both humoral and cell-mediated immune responses potentially contributing to protection. Infections can be asymptomatic but still contribute to chronic sequelae; thus, identification of biomarkers for individuals at high risk for disease is urgently needed (24–26). With the development of high-throughput molecular profiling technologies, classical algorithms that emphasize *P* values of individual features to identify impactful factors provide low classification accuracy. ML algorithms that “learn” data patterns efficiently, potentially enhancing prediction accuracy, are becoming increasingly important. However, study designs frequently predetermine ML algorithms, arbitrarily choose feature selection procedures, construct and evaluate prediction on static data, and neglect biological interpretation of biomarkers. To surmount these challenges, we established a resampling-based ML pipeline for identification of optimal ML methods and biomarkers. This pipeline provides a reusable framework for biomarker discovery and can be generalized to any predictive modeling for a health outcome.

This pipeline features multiple strengths. First, it systematically searches for candidate algorithms from over 200 ML algorithms, circumventing bias in selection. Second, it uses permutation-based feature importance ranking. The feature importance describes which features contribute most to the outcome prediction, which is critical not only for feature selection but also for biological interpretation of biomarkers. A challenge when ranking importance is that correlated features can lead to the selection of one feature while correlated features are neglected. The permutation-based identification of important features, which randomly shuffles each feature and computes the change in the model’s performance, can overcome this drawback. Third, it identifies biomarkers by a resampling-based recursive feature elimination (RFE) procedure, which evaluates the impact of ranked features on model fit starting with all features and followed by backward selection, enabling capture of all important features. A potential drawback of RFE is “overfitting,” but incorporating resampling in feature selection can help to address this issue. Fourth, this pipeline leverages a stratified resampling strategy, which ensures that the training and test sets have approximately the same percentage of positive and negative outcome groups as the complete data, avoiding sampling bias. Although resampling provides better estimates, a potential disadvantage is that computational time can be relatively high. It may also appear confusing that different lists of important features may be generated in each resampled data set. However, this provides a more probabilistic assessment of feature importance and prevents the extreme value of performance due to point estimation from a single fixed data set.

When the 4 ML algorithms in this study were compared, none demonstrated a universal best performance across different outcomes. Naive Bayes had the best AUROC for ascension compared to the other models, while random forest and xgbLinear had better performance for incident infection. However, the nonparametric complex models such as the tree-based algorithms, including random forest and xgbLinear, were more susceptible to overfitting, due to flexibility in model training. For example, anti-CT110 frequencies were not different in the F/U+ and F/U− groups (*P* = 0.87 [Fig. S1]), suggesting that this antibody is an irrelevant IgG, but it was selected as a biomarker for incident infection by random forest and xgbLinear. In addition to the deployment of techniques to prevent complex models from overfitting, we recommend leveraging different ML algorithms to capture consensus biomarkers, since there is no one-size-fits-all method.

Feature selection is one of the most critical stages of an ML pipeline. Comparison of two well-established feature selection methods revealed that RFE had better performance than Boruta for this data set. However, their performances depend heavily on data and their variables’ distribution. We recommend exploring both methods and determining which one works better for the training data set.

One of the drawbacks of ML algorithms is a lack of functional annotation. Most ML models are uninterpretable and are largely data driven. Thus, to identify ideal biomarkers of a disease, it is critical to follow up on prediction models with further hypothesis testing and external validation using independent large cohorts. We applied causal mediation analysis to facilitate hypothesis generation and testing with the goal of enabling functional interpretation of biomarkers. The causal mediation analysis revealed that anti-Pgp3 and anti-CT123 reduced the risk of ascension through cervical *Ct* burden-dependent and/or -independent effects. Pgp3 is a plasmid-encoded virulence factor. It has been reported to play roles in Chlamydia host cell adhesion and invasion (27) and neutralization of the antichlamydial activity of an antimicrobial peptide (28). It is highly immunogenic (29) and promotes *Ct* infectivity and pathogenicity (30). The antibody-mediated inhibition of Pgp3’s functions could impair *Ct* invasion. CT123 (AccB) is a biotin carboxyl carrier protein of acetyl-CoA carboxylase, the first committed enzyme of the fatty acid synthesis pathway. Fatty acid synthesis is essential for *Ct* proliferation within its host, since *Ct* has a reduced genome and relies on *de novo* fatty acid and phospholipid biosynthesis to produce its membrane phospholipids. However, anti-Pgp3 and anti-CT123 were very weak biomarkers, with median prediction AUROC only around 0.6, suggesting that detection of these binding antibodies is insufficient for predicting absence of ascension.

For incident infection, seropositivity consensus biomarkers, including anti-CT443 (OmcB, outer membrane complex protein), anti-CT681 (MOMP), and anti-CT828 (NrdB, a virulence-associated type III secretion system protein) occurred more often in F/U+ women, suggesting that detection of binding antibodies to these proteins fails to associate with protection from reinfection or control of a repeat infection. B cells and antibody responses can complement T cell-mediated protection against *Ct* but are secondary to essential cell-mediated adaptive T cell responses for resolving *Ct* infection and preventing reinfection (31–33). All three antigens were immunodominant for antibody in this cohort and others (13, 34). It is likely that detection of larger amounts of antibody in infected women reflects compromised development of CD4 T cell responses that limit burden and duration of infection, leading to increased antigen exposure and subsequent antibody production.

In addition, we previously identified anti-Pgp3 and anti-CT443 as the antibodies with the greatest impact on ascension and incident infection, respectively, by logistic regression (13), consistent with our findings obtained by using ML methods in this study. However, the low prediction accuracies of these previously identified most significant features reiterate the disadvantage of focusing on *P* values of individual features for impactful factors as predictors. Significant association of a feature with the outcome may not necessarily imply that it is a good biomarker. Association tests examine group differences among populations, while predictions assess the correctness of classifying unseen individuals. The distinction has been elaborated by simulated examples and theoretical explanation (35, 36). For instance, a continuous feature can be significantly associated with a binary outcome but have low prediction accuracy, because the feature distributions in cases and controls partially overlap. In addition, a binary feature can be significant due to a small group of subjects in the population. A pipeline that systematically searches for biomarkers and comprehensively evaluates the prediction performance has great advantages.

Another issue is to determine the optimal sample size, which depends on multiple factors, including the complexity of data, the number of features, the level of noise in the data, the machine learning model type, and the desired accuracy. Several methods have been proposed to estimate the sample size for ML analysis (37–39), which nevertheless set an upper limit of error rate given a sample size only for certain model types or *post hoc* fitting of a learning curve. Currently, there is no formulaic algorithm to determine the number of samples for a given ML model. Furthermore, the data quality is more important than the quantity, so performance will not improve with increasing sample size if the data are noisy (40). At a practical level, prior feasibility or a pilot study may give some guidance on anticipated data quality. However, new statistical approaches that can aid sample size calculations are also needed.

In addition, our findings suggest that the predictive performances for ascension and incident infection using *Ct* protein-specific serum IgGs and clinical factors were undesirable. Human and animal studies report that T cells which produce gamma interferon (IFN-γ) are key mediators of host defense against *Ct* (41–44). Future studies are warranted to examine antigen-specific responses mediated by immune T cells and investigate their potential as biomarkers for *Ct* ascension and incident infection.

## MATERIALS AND METHODS

### Study population and data collection.

Data used in this analysis were from 232 women recruited into the T cell Response Against Chlamydia (TRAC) study (12). Data from 10 women were excluded from further analysis due to ambiguous testing results for chlamydial infection. Participants returned for follow-up visits at 1, 4, 8, and 12 months after enrollment, when data and specimen collections were repeated but no endometrial biopsy was performed. Women testing positive for chlamydial infection during follow-up were treated with azithromycin. Women who did not complete 3 follow-up visits were excluded from reinfection analysis.

At enrollment, demographics, information regarding sexual exposure, and clinical data and microbiology data from cervical swab and endometrial biopsy were obtained (12, 13). C. trachomatis-specific serum IgG obtained from the study participants was profiled by whole-proteome microarrays, and the 121 most reactive proteins were identified as previously described (13). Briefly, whole-proteome microarray slides comprising 895 Chlamydia proteins were first used to analyze serum pools from TRAC enrollment samples. Antigen-specific IgG binding seropositivity was determined by neighborhood averaging (45). The 118 most reactive proteins identified using serum pools on whole-proteome microarrays and three proteins frequently recognized by T cells from TRAC participants were selected for minimized microarrays, which yielded 121 proteins for individualized recognition profiling.

### Identification of 4 candidate ML algorithms using SIMON.

Positivity for antibodies to 121 chlamydial proteins and previously identified important risk factors associated with ascension (Neisseria gonorrhoeae infection and oral birth control pills) and incident infection (age, gonorrhea, Chlamydia infection at enrollment, and sex with new, uncircumcised, or infected partners) (12) were examined for their predictive performance regarding ascension and incident infection by ML algorithms. Analysis was stratified to assess incident infection for women uninfected or infected at enrollment. With our goal of identifying *Ct*-specific antibodies as biomarkers for ascension and/or reinfection, 55 proteins with low frequency of antibody reactivity (recognition by <10% of the cohort) and 3 additional proteins with low reactivity levels (average fold change intensity expression above negative controls in reactive women, <1.5) were filtered from further analysis.

We first used SIMON to systematically screen for candidate machine learning algorithms. SIMON (17) is an open-source software with an automated ML system that compares results from 215 different algorithms. To estimate classification accuracy, SIMON randomly partitions the data set into 2/3 training and 1/3 test sets by stratified split to maintain a balanced distribution of positive and negative outcome groups in training and test sets (17) using the function createDataPartition from the Caret package. All ML algorithms were processed in an automated way through the Caret library (46). Each model was developed using 10-fold cross-validation in the training set. The performance of each model was evaluated on the test set, which had been removed before model training. Feature selection was performed using the R package Boruta (7).

### Comparison of the performance of 4 candidate ML algorithms with the RFE and Boruta feature selection methods.

Next, we used the resampling-based recursive feature elimination (RFE) procedure to compare the prediction performance of the 4 candidate algorithms, to identify the optimal ML algorithm and important classifiers as biomarkers. RFE is a backward feature selection method, which searches for a subset of features by starting with all features in the training data set and then removing features until a specified number remained. This is achieved by fitting the given ML algorithm used in the core of the model, ranking features by importance, discarding the least important features, and refitting the model. This process is repeated until a specified number of features remain. To compare the performance of ML algorithms, the data were randomly partitioned into 2/3 training and 1/3 testing data 100 times to generate 100 training and test data sets, with balanced class distribution of positive- and negative-outcome groups. In each training data set, the importance of each feature was evaluated and ranked.

The variable importance score is calculated using function varImp from the R package caret (v6.0-88). For the random forest method, permuted conditional variable importance (R function cforest from the package party) is used to calculate variable importance score; it could deal with correlation between predictors and the situation while different types of predictor exist. For other methods, the importance of each predictor is evaluated individually using model independent metrics. The AUROC of each predictor is used as the measure of variable importance.

All ranked features were defined as nested subsets of features: *F_n_* (all features) ⊃ *F_n_*_−1_ (top *n*−1 ranked features) ⊃ *F_n_*_−2_ (top *n*−2 ranked features)…, and the best subset of features with the smallest generalization error was determined by 10-fold cross validation and by varying a single parameter, i.e., the number of features. The best subset of features was used to generate a prediction model in the training data and was evaluated in the corresponding independent test data to determine predictive performance. This recursive feature selection procedure was conducted using the function rfe from the caret package (v.6.0.81) in R. We repeated the procure 100 times using all resampling data sets. The performances of prediction with respect to AUROC, accuracy, sensitivity, and specificity in 100 data sets were visualized by box plots. The median of performances from 100 data sets was calculated using true median, which can deal with ties, implemented in the R package fmsb. Medians of different groups were compared using Mood’s median test.

After the optimal ML algorithm was determined, the variable importance scores across 100 training sets were averaged, and the ranks of features were recalculated for the generalization error, which was assessed using 10-fold cross-validation in each training data and averaged across all 100 training data sets. The optimal number of features which had the smallest average generalization error was selected as the final set of biomarkers.

Next, we used the resampling-based Boruta feature selection procedure to compare the prediction performance of the 4 candidate algorithms. Boruta generates a randomized copy of all features, combines them with the original features, then builds random forest on the merged data set, and finally compares the original features with the randomized features to determine feature importance. Only features having higher importance than the randomized ones are considered important. We used the same 100 training and testing sets with balanced class distribution of positive and negative outcomes as in the RFE procedure and used the R package Boruta to implement this feature selection method. We constructed a total of 500 trees to identify the important features. We subsequently applied each of 4 ML algorithms on the selected important features to generate a prediction model in the training data and evaluate predictive performance in the corresponding independent test data.

### Causal mediation analysis and biomarker interpretation.

We considered ascending infection status (Endo+ versus Endo−) as a binary outcome, and we defined Chlamydia cervical burden as a mediator and antibody as an exposure. We investigated the association of antibody with endometrial infection by a causal mediation algorithm (19) (Fig. S2), which was described in detail previously (15). Using causal mediation, the relationship between antibody and ascension is decomposed into a direct link and an indirect link. The causal mediation effect represents the indirect effect of the exposure (antibody) on the outcome (ascension) through the mediating variable (bacterial burden), whereas the direct effect is the effect of the exposure (antibody) on the outcome (ascension) that is independent of the mediator (burden). The total effect is the sum of the indirect and direct effects. This mediation test was conducted using the R package mediation (version 4.5.0).

The risks of ascension and incident infection in women positive for antigen-specific antibodies compared to those negative to biomarkers were visualized by bar chart and tested by chi-square test for significance.

### Code availability.

The R codes for our ML analysis pipeline and causal mediation analysis in this paper are available at https://github.com/lcw68/chlamydia-ML.

### Data availability.

The original contributions presented in the study are included in the article. Further inquiries can be directed to the corresponding author.
