# Supplementary material for: A Machine Learning-Based Analytic Pipeline Applied to Clinical and Serum IgG Immunoproteome Data To Predict Chlamydia trachomatis Genital Tract Ascension and Incident Infection in Women
Source: Microbiol Spectr. 2023 Jun 15;11(4):e04689-22. doi: 10.1128/spectrum.04689-22 (PMC10434056; doi:10.1128/spectrum.04689-22)
Supplement: Supplemental file 1 — Supplemental material. Download spectrum.04689-22-s0001.docx, DOCX file, 0.03 MB [file spectrum.04689-22-s0001.docx]

**Supplementary Table 1. Top 4 best ML algorithms from SIMON**

| Ranks | Ascending infection | Reinfection in infected women at enrollment | Reinfection in uninfected women at enrollment |
| --- | --- | --- | --- |
| 1 | **eXtreme Gradient Boosting with linear booster*** | oblique random forest using PLS as splitting model | **Naïve Baiyes** |
| 2 | C5.0 | **Naïve Baiyes** | Support Vector Machines with Radial Basis Function Kernel |
| 3 | C5.0Cost | Multilayer perceptron | Spiking neural networks |
| 4 | C5.0Tree | **K-Nearest Neighbors** | **Random Forest** |
| 5 | **Random Forest** | Bayesian generalized linear models | Partition around medoids |
| 6 | Parallel Random forest | **eXtreme Gradient Boosting with linear booster** | **K-Nearest Neighbors** |
| 7 | Kernel Partial Least Squares Regression | Linear Support Vector Machines with Class Weights | Classical SIMCA |
| 8 | Linear Distance Weighted Discrimination | Boosted Logistic Regression | Random Forest Rule-Based Model |
| 9 | **Naïve Baiyes** | Gradient Boosting Machine | Boosted Linear Model |
| 10 | **K-Nearest Neighbors** | **Random Forest** | **eXtreme Gradient Boosting with linear booster** |

*****Bolded methods were consistently selected as the top ML algorithms across three outcomes.
