# Supplementary figures and images for: A Machine Learning-Based Analytic Pipeline Applied to Clinical and Serum IgG Immunoproteome Data To Predict Chlamydia trachomatis Genital Tract Ascension and Incident Infection in Women
Source: Microbiol Spectr. 2023 Jun 15;11(4):e04689-22. doi: 10.1128/spectrum.04689-22 (PMC10434056; doi:10.1128/spectrum.04689-22)

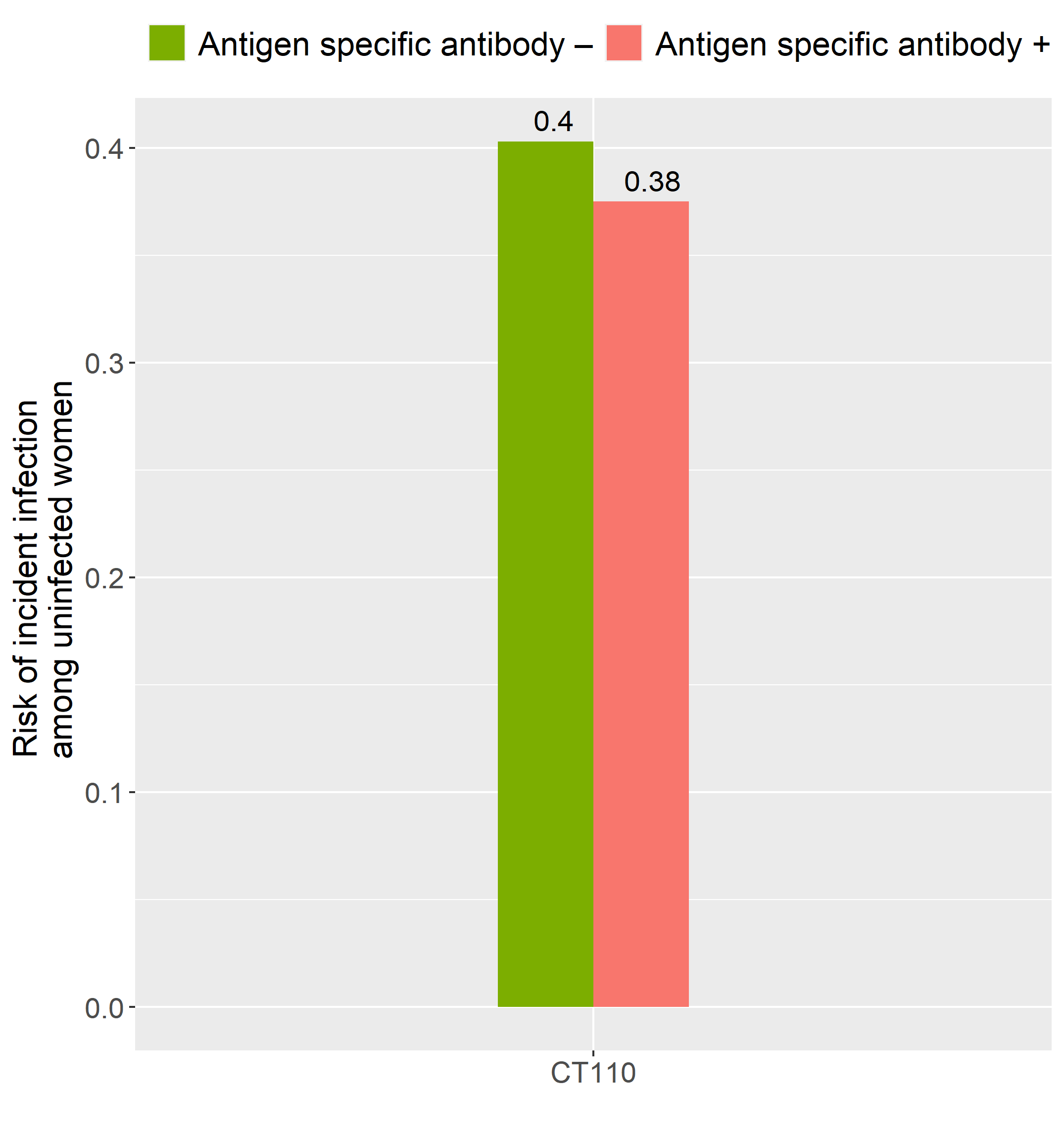

Supplement: Supplemental file 2 — Supplemental material. Download spectrum.04689-22-s0002.tif, TIF file, 14.1 MB [file spectrum.04689-22-s0002.tif]

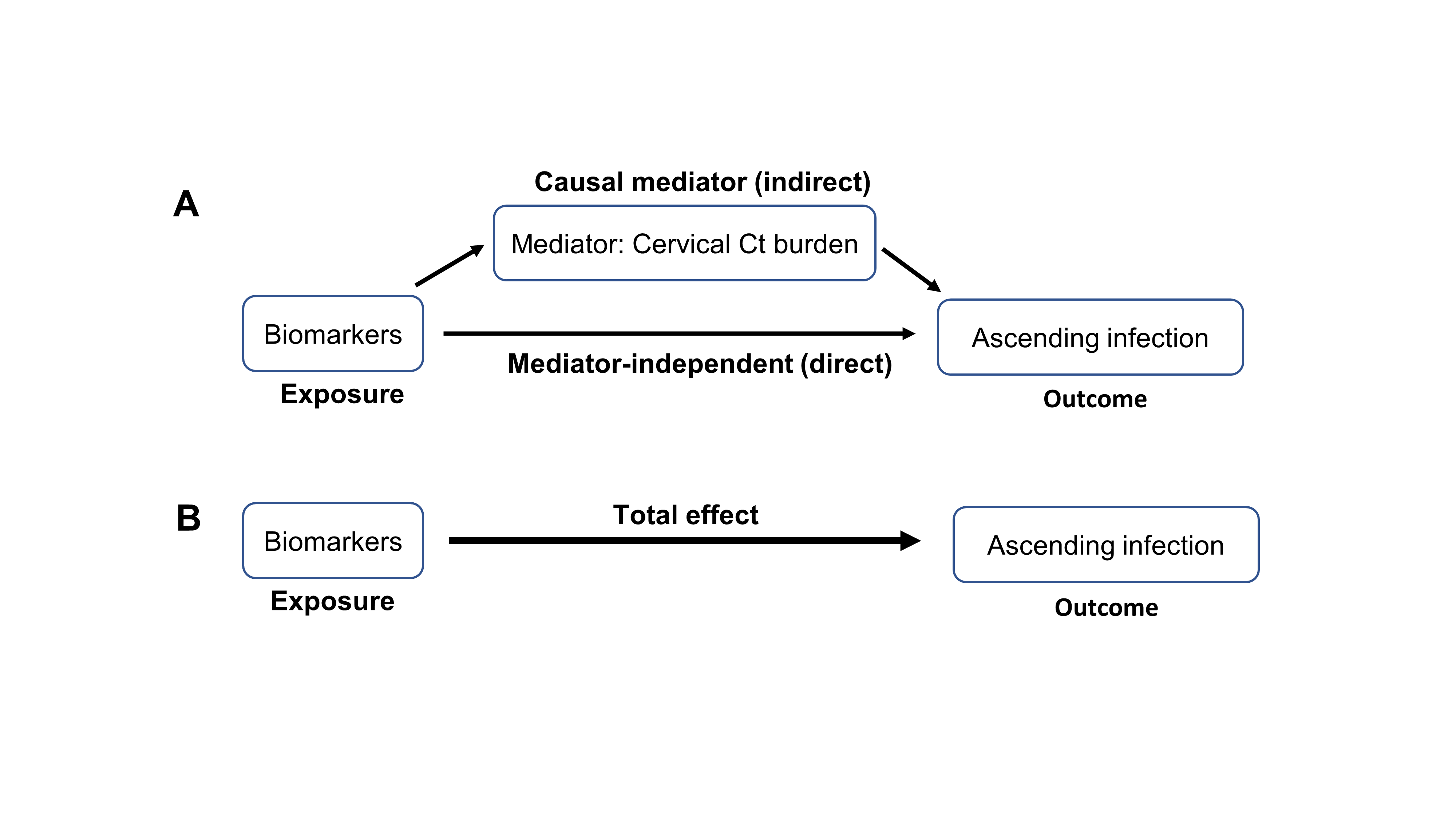

Supplement: Supplemental file 3 — Supplemental material. Download spectrum.04689-22-s0003.tif, TIF file, 0.5 MB [file spectrum.04689-22-s0003.tif]
