# Supplementary material for: A Machine Learning-Based Analytic Pipeline Applied to Clinical and Serum IgG Immunoproteome Data To Predict Chlamydia trachomatis Genital Tract Ascension and Incident Infection in Women
Source: Microbiol Spectr. 2023 Jun 15;11(4):e04689-22. doi: 10.1128/spectrum.04689-22 (PMC10434056; doi:10.1128/spectrum.04689-22)
Supplement: Supplemental file 4 — Supplemental material. Download spectrum.04689-22-s0004.docx, DOCX file, 0.02 MB [file spectrum.04689-22-s0004.docx]

**Supplementary Fig 1**. Seropositivity to CT110 was not associated with the risk of incident infection among infected women at enrollment, (P=0.87 by chi-square test). The numbers on the top of each bar indicate the incident risk of incident infection among antibody negative and positive response groups.

**Supplementary Fig 2**. (A) Diagram of causal mediation analysis of the relation of biomarkers (Exposure) cervical *C. trachomatis* burden (Mediator) and ascending infection (Outcome). (B) The sum of mediator-dependent and –independent, effects equal the total effect.
